# CGP17Pat: Automated Schizophrenia Detection Based on a Cyclic Group of Prime Order Patterns Using EEG Signals

**DOI:** 10.3390/healthcare10040643

**Published:** 2022-03-29

**Authors:** Emrah Aydemir, Sengul Dogan, Mehmet Baygin, Chui Ping Ooi, Prabal Datta Barua, Turker Tuncer, U. Rajendra Acharya

**Affiliations:** 1 Department of Management Information Systems, Management Faculty, Sakarya University, Sakarya 54050, Turkey; emrahaydemir@sakarya.edu.tr; 2 Department of Digital Forensics Engineering, College of Technology, Firat University, Elazig 23119, Turkey; turkertuncer@firat.edu.tr; 3 Department of Computer Engineering, College of Engineering, Ardahan University, Ardahan 75000, Turkey; mehmetbaygin@ardahan.edu.tr; 4 School of Science and Technology, Singapore University of Social Sciences, Singapore 599494, Singapore; cpooi@suss.edu.sg; 5 School of Business (Information System), University of Southern Queensland, Toowoomba, QLD 4350, Australia; prabal.barua@usq.edu.au; 6 Faculty of Engineering and Information Technology, University of Technology Sydney, Sydney, NSW 2007, Australia; 7 Ngee Ann Polytechnic, Department of Electronics and Computer Engineering, Singapore 599489, Singapore; aru@np.edu.sg; 8 Department of Biomedical Engineering, School of Science and Technology, SUSS University, Singapore 599491, Singapore; 9 Department of Biomedical Informatics and Medical Engineering, Asia University, Taichung 413, Taiwan

**Keywords:** cyclic group of prime order pattern, schizophrenia detection, EEG classification, NCA, kNN, machine learning

## Abstract

Background and Purpose: Machine learning models have been used to diagnose schizophrenia. The main purpose of this research is to introduce an effective schizophrenia hand-modeled classification method. Method: A public electroencephalogram (EEG) signal data set was used in this work, and an automated schizophrenia detection model is presented using a cyclic group of prime order with a modulo 17 operator. Therefore, the presented feature extractor was named as the cyclic group of prime order pattern, CGP17Pat. Using the proposed CGP17Pat, a new multilevel feature extraction model is presented. To choose a highly distinctive feature, iterative neighborhood component analysis (INCA) was used, and these features were classified using k-nearest neighbors (kNN) with the 10-fold cross-validation and leave-one-subject-out (LOSO) validation techniques. Finally, iterative hard majority voting was employed in the last phase to obtain channel-wise results, and the general results were calculated. Results: The presented CGP17Pat-based EEG classification model attained 99.91% accuracy employing 10-fold cross-validation and 84.33% accuracy using the LOSO strategy. Conclusions: The findings and results depicted the high classification ability of the presented cryptologic pattern for the data set used.

## 1. Introduction

Schizophrenia is a serious mental illness where patients have difficulty distinguishing between what is real and what is not [1,2]. Schizophrenia is a chronic brain disorder that affects a person’s thoughts, feelings, and behavior [3]. People with schizophrenia often experience symptoms such as hallucinations, delusions, abnormal behavior, and disorganized speech [4,5]. Although the symptoms come and go, schizophrenia significantly affects the social lives, education, and professional performance of the affected individuals [6].

Schizophrenia is a global disorder [7] with a prevalence of approximately 1% worldwide, and it is reported that 20 million people are affected by schizophrenia [8]. In addition, studies have shown that schizophrenia is more common in men than women [9,10]. The disorder usually starts between 18 and 25 years in men and between 25 and 35 years in women [11]. The causes of schizophrenia are still unknown, but researchers have observed that genetics, brain chemistry, and environment may be associated with the development of the disorder [12,13]. Moreover, psychological factors are also known to trigger this disorder. Schizophrenia is treatable with medication and psychosocial support [14]. Without treatment, people with schizophrenia can develop other mental health disorders and significant health problems. It is thus essential to establish a correct diagnosis and early treatment of the disease.

Schizophrenia (SZ) is diagnosed from the patient’s symptoms and a specialist’s opinion in mental health. In addition, techniques such as magnetic resonance imaging, computed tomography, and EEGs can also be used in the diagnostic phase [15,16]. In short, there is no single test method to diagnose SZ. These days, machine learning techniques are actively used to automatically interpret EEG signals [17]. Due to the fact of these machine learning techniques, EEG data collected from patients are automatically classified, permitting early diagnosis of various diseases. One of these diseases is SZ, and there are various studies on automatic SZ classification in the literature as summarized in Table 1.

Various data sources, such as EEG signals and MRI imaging, have been used to support schizophrenia diagnosis via machine learning. As shown in Table 1, EEG signals are some of the most commonly used medical data for schizophrenia diagnosis. Table 1 also shows the various EEG signal-based schizophrenia detection models that were developed using hand-crafted, feature-based deep learning models. The deep learning models attained high classification performances but had high computational complexities. Moreover, most of the EEG signal-based schizophrenia detection models did not use LOSO cross-validation [33]. The signals were segmented, which may cause overfitting with other validation techniques. On the other hand, real classification performance can be accurately calculated using LOSO cross-validation. Therefore, LOSO cross-validation is a critical validation technique, and in this work, we used LOSO cross-validation in our proposed model.

In this paper, two validation techniques (i.e., 10-fold cross-validation and LOSO validation) were used, and high classification accuracies were obtained with our developed model. Our proposed model can automatically classify EEG signals collected from schizophrenia patients and healthy individuals. The new feature extractor (i.e., CGP17Pat) was also tested on an open-access schizophrenia data set with much success.

Feature engineering is one of the most important machine learning and classification issues. By deploying feature engineering methodologies, hand-crafted feature engineering models have been developed. In this work, we employed a cryptologic structure to propose a new nonlinear pattern. Cryptographic systems generally use finite groups that have been generated using various algorithms. The most popular finite group creation model is multiplication-based group creation. The prime numbers have been used to create cyclic finite group and is named the cyclic group of prime (CGP) order [34,35]. Using the values of the group and a prime number, unique vectors are generated, and these vectors can be used to create permutation or substitution boxes. In this work, using these nonlinear values by generating CGP, a new local binary pattern, such as a signal descriptor, was generated. A local binary pattern and versions of it, generally, used a pattern to generate features. Using CGP with modulo 17, eight different patterns were created, and all of these patterns were applied to the signal to generate a feature vector. Thus, the proposed feature extraction function was named CGP17Pat.

Schizophrenia is a serious mental disorder, and diagnosing schizophrenia is not easy. Constant follow ups with suspected cases are required before a diagnosis can be made. Moreover, early diagnosis and treatment are vital for a better prognosis. Hence, to simplify the diagnosis process of schizophrenia, an automated EEG-based diagnosis model is proposed. Our machine learning method uses CGP17Pat as the feature extractor. A successful machine learning method needs an effective feature extractor, a feature selection function to choose the most discriminative ones, and an appropriate classifier. Our proposed model extracted 2048 features using eight patterns of the presented CGP17Pat. The MAP decomposer was used to create high-level features, while INCA [36] was employed to choose the top features. kNN [37] was employed to generate channel-wise results using 10-fold cross-validation and LOSO. Finally, iterative hard majority voting created the general results. The objectives of this study were (i) to show the feature generation ability of the proposed CGP17Pat, (ii) develop an automated schizophrenia detection model using EEG signals with a low time burden, and (iii) to analyze the schizophrenia detection ability using each EEG channel.

A new generation hand-modeled EEG signal classification method is proposed in this research, and the novelty of this research is the CGP17Pat. CGP has generally been used for cryptographic engineering models, since it is a finite group creator. CGP and 17 (a prime number) were used in this work to present a new feature extractor. The contributions of this work are given as below.

(i) A new one-dimensional feature extraction function using a cryptographic model is proposed. The main aim of this feature extractor was to suggest a nonlinear local feature extractor, and this feature extractor is a local binary pattern (LBP) feature extractor. Therefore, the proposed CGP17Pat generates textural features. The informative feature generation ability of CGP17Pat is demonstrated using EEG signals; 

(ii) The CGP17Pat is the main feature extraction function of this model. An effective feature selector (i.e., INCA) was employed to decrease the number of features, and a shallow/conventional classifier (i.e., kNN) was deployed to obtain the classification results. Furthermore, two validations methods (i.e., 10-fold cross-validation and LOSO) were used to validate the robustness of the CGP17Pat-based EEG signal classification method. A schizophrenia data set with 19 channels was analyzed in this work.

## 2. Materials and Methods

### 2.1. Materials

This study used freely available data from a public repository created by Olejarczyk and Jernajczyk [18,19]. The 10/20 EEG montage methodology was used to collect the EEG signals. It consists of EEG recordings from 14 schizophrenic patients and 14 healthy subjects from the Institute of Psychiatry and Neurology in Warsaw (excluding brain disordered patients). To choose the control group, gender and age criteria were considered. The patients included in this study consisted of those above 18 years of age with a diagnosis of F20.0 in the ICD-10 category. These patients did not use any drugs at least seven days before data recording. Pregnant patients, patients with organic brain pathology, severe neurological diseases, the presence of a general medical condition, and persons under 18 years of age were excluded from the study. In addition, early-stage patients, such as those exhibiting their first attack, were not included in the study. The recordings were for an average of 15 min with eyes closed and in a resting state. The electrodes were placed according to the 10/20 system. Data were collected from 19 channels (i.e., Fp1, Fp2, F7, F3, Fz, F4, F8, T3, C3, Cz, C4, T4, T5, P3, Pz, P4, T6, O1, and O2) at a sampling rate of 250 Hz. Further information on the collected EEG data is given in the Table 2 below.

### 2.2. Method

**Figure 1:**
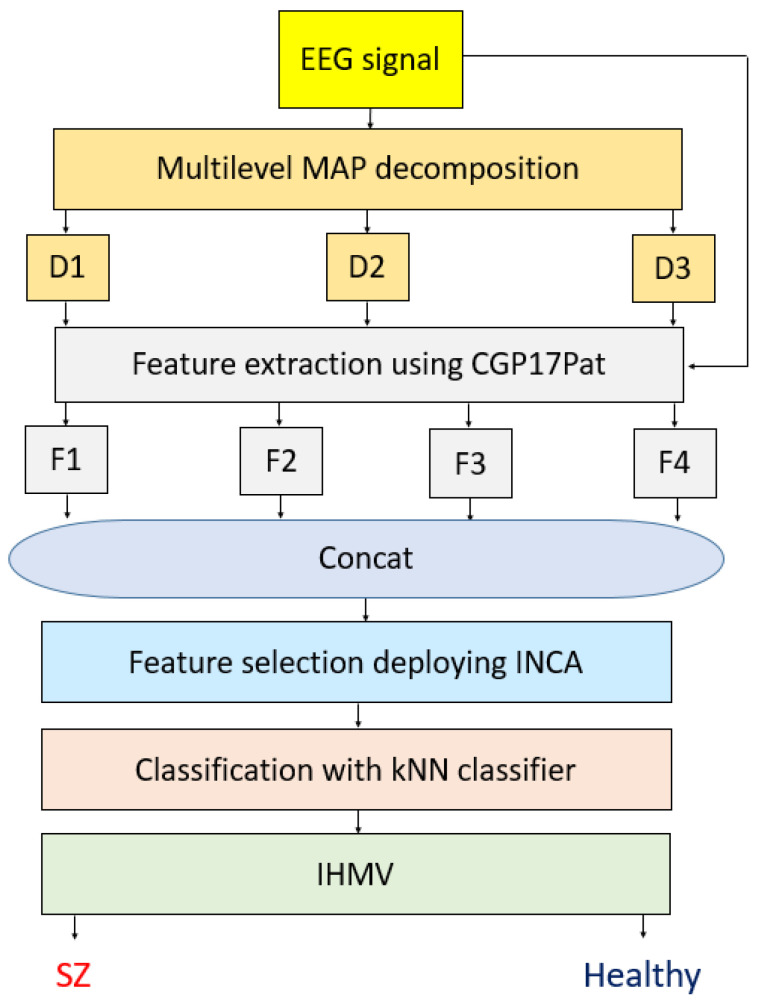
Schematic diagram of the proposed CGP17Pat-based schizophrenia detection model.

The main aim of the proposed model was to show the automatic detection ability of a novel nonlinear feature extraction model using the CGP model. CGP has been used to create finite cyclic groups. A basic EEG signal classification hand-modeled method has been presented to detect schizophrenia automatically. Our method contains textural feature generation using the presented CGP17Pat, the most informative features chosen using INCA, classification, and iterative hard majority voting phases. The schematic summarization of our model is illustrated in Figure 1.

In this model, EEG signals are decomposed using the multilevel MAP function. In this function, 2-, 4-, and 8-sized overlapping blocks are used to generate decomposed signals (i.e., D1, D2, and D3). In the feature extraction phase, the proposed CGP17Pat is employed for each decomposed signal and raw EEG signal. Therefore, four vectors (i.e., F1, F2, F3, and F4) are created. The length of each feature vector generated is 2048. These feature vectors are merged in the feature merging step (concat), and a feature vector with a length of 8192 (=2048 × 4) is created. INCA chooses the most informative features, and kNN calculates the channel-wise results. Iterative hard majority voting is deployed to generate/calculate the general classification performance using 19 channels.

#### 2.2.1. Feature Creation

The first phase is feature vector creation. Before feature extraction, each EEG signal is divided into segments of 10 s. Then, a mathematical function/method is used to create the feature generator automatically. The used mathematical function is CGP. Here, 17 (Z_17_) is selected as the prime order. By utilizing 17, eight cyclic groups are obtained. The general function of the CGP is given in Equation (1):

(1) $$a^{i}\;\left(mod\;p\right),\;a\in \left\{2,3,\ldots ,p-1\right\},\;i\in \left\{1,2,\ldots ,p-1\right\}$$

By deploying this equation (Equation (1)), cyclic groups are generated. Herein, p defines the prime number, a represents the used number to create a group, and i is the exponent. By deploying 17 and Equation (1), eight cyclic groups are generated, and the generated cyclic groups are tabulated in Table 3.

**Figure 2:**
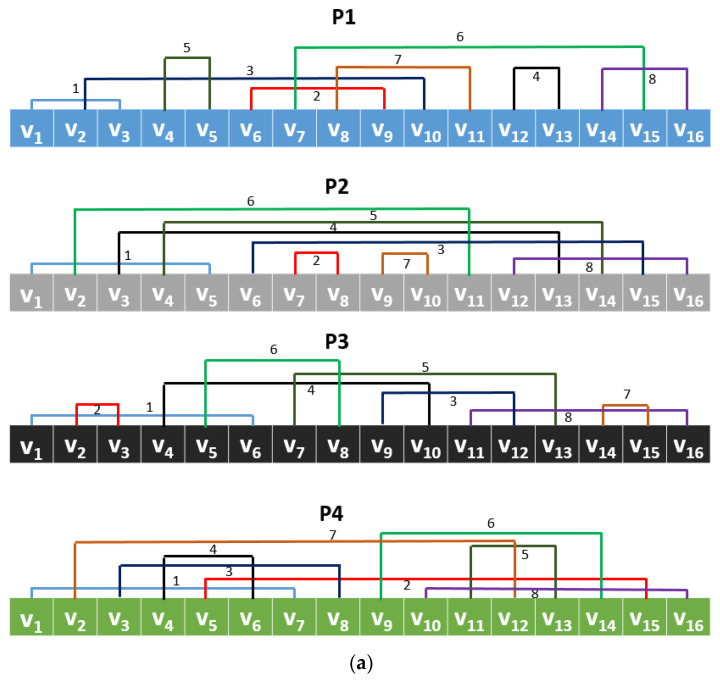

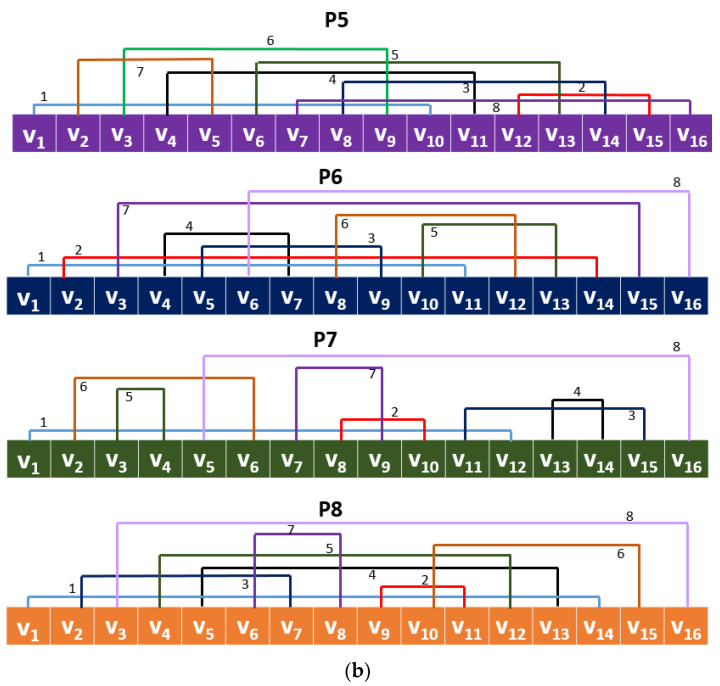
The created eight patterns using CGP17, where each pattern is named as P; by using each pattern, 256 features are extracted and our presented CGP17Pat uses these eight patterns together: (**a**) patterns 1–4; (**b**) patterns 5–8.

By deploying these values (Table 3), a multiple pattern center symmetric feature extraction function is presented (CGP17Pat). The created eight patterns using Table 3 are also demonstrated in Figure 2.

**Figure 3:**
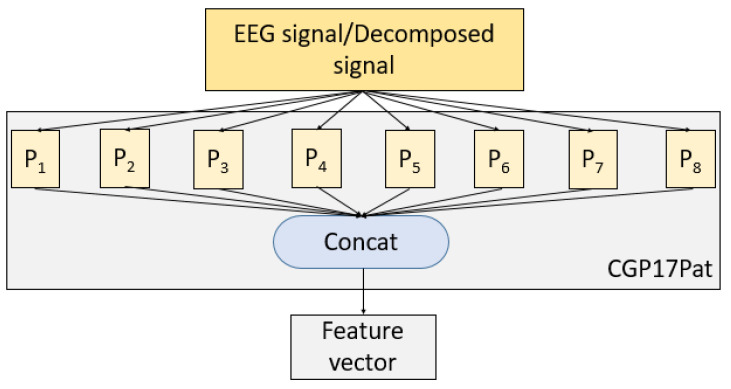
The graphical summarization of the presented CGP17Pat. Here, P denotes patterns (see Table 2), and each pattern extracts 256 features. Then, these feature vectors are merged, and 2048 (=256 × 8) features are created.

To better explain the presented CGP17Pat feature extractor, a schematic denotation is shown in Figure 3.

MAP is used to create textural features at both low and high levels. The details of our presented MAP and CGP17Pat-based model are as below.

Step 1: Create decomposed signals using the MAP method. The mathematical definition of the MAP is denoted in Equation (2):

(2) MAP(a,b)=max(|bl|), bl(j)=a(i+j−1)j∈{1,2,…,b}, i∈{1,b,…,leng−b+1}

Herein, MAP(.,.) is the maximum absolute pooling function, a is the input signal, b defines the length of the used non-overlapping block (bl), leng represents the length of the used EEG signal, |.| function is the absolute value calculation function, and i,j defines indexes.

The raw EEG signal is divided into 2-, 4-, and 8-sized non-overlapping blocks, and three decomposed signals are created. Decomposed signal creation is defined in Equation (3).

(3) $$D^{t}=MAP\left(ES,2^{t}\right),\;t\in \left\{1,2,3\right\}$$

Herein, $D^{t}$ defines the *t*th decomposed signal.

Step 2: Extract features from decomposed signals and the raw schizophrenia EEG signal deploying the presented CGP17Pat.

(4) $$F^{1}=CGP17Pat\left(Sgnl\right)$$

(5) $$F^{t+1}=CGP17Pat\left(D^{t}\right),\;t\in \left\{1,2,3\right\}$$

where *CGP17Pat*(.) is the introduced feature extraction function, and *F* defines the generated features’ vectors with a length of 2048. Our proposed function (*CGP17Pat*(.)) was deployed with raw EEG signals (*Sgnl*) and decomposed signals to extract features. Our proposed textural feature extraction function details are depicted in sub-steps (Step 2.1–2.4).

Step 2.1: Create overlapping blocks with a length of 16.

(6) $$b^{i}\left(j+1\right)=Sgnl\left(i+j\right),\;i\in \left\{1,2,\ldots ,leng-15\right\},\;j\in \left\{0,1,\ldots ,15\right\}$$

Herein, $b^{i}$ is the *i*th overlapping block.

Step 2.2: Generate binary features using the signum function.

(7) $$bit^{h}\left(k\right)=sign\left(b^{i}\left(G^{h}\left(k\right)\right),b^{i}\left(G^{h}\left(17-k\right)\right)\right),h\in \left\{1,2,\ldots ,8\right\},k\in \left\{1,2,\ldots ,8\right\}$$

(8) $$sign\left(t,l\right)=\{\begin{array}{c} 0,t-l<0\\ 1,\;t-l\geq 0 \end{array}$$

Herein, $bit^{h}$ is the *h*th bit group with a length of eight, and we generated eight-bit groups deploying the signum (sign(.,.)) function; $G^{h}$ defines the *h*th cyclic group (see Table 2).

Step 2.3: Generate eight map signals deploying the created bits (binary features).

(9) $$map^{h}\left(i\right)=\overset{8}{\underset{k=1}{\sum }}bit^{h}\left(k\right)\times 2^{h-1}$$

Herein, $map^{h}$ is the *h*th map signal.

Step 2.4: Extract a histogram of each map signal and merge these histograms. The length of each histogram is 256. Therefore, the proposed CGP17 creates 256 × 8 = 2048 features from each EEG segment.

Step 3: Merge/concatenate the generated textural vectors (i.e., $F^{1},\;F^{2},\;F^{3},\;\mathrm{and}\;F^{4}$) to create a merged feature vector with a length of 8192.

(10) $$ft\left(h+2048\times \left(t-1\right)\right)=F^{t}\left(h\right),\;h\in \left\{1,2,\ldots ,2048\right\},\;t\in \left\{1,2,3,4\right\}$$

where ft merges the feature vector that has a length of 8192.

#### 2.2.2. Feature Selection

The INCA (an improved/developed version of the NCA selector) feature selector is applied to the most distinctive features from the generated 8192 features in the feature selection phase. The NCA is a weight-based feature selector and is a kNN-like function. To increase the automatic feature selection ability of the NCA, INCA was proposed by Tuncer et al. [38,39]. INCA uses two parameters, and these parameters are named iteration range and loss value calculator. The iteration range is used to decrease the computational complexity of the INCA. In this study, the iteration/loop was initialized at 100 and finished at 1000.

Step 4: Apply the NCA to the generated features and obtain the qualified indexes.

Step 5: Choose the most valuable/meaningful from 100 to 1000 features using the generated qualified indexes. By using this iterative feature selection, 901 feature vectors are chosen.

Step 6: Find the feature vector with the minimum loss value.

Step 7: Select the most appropriate feature vector according to Step 6.

#### 2.2.3. Classification

In the classification phase, a conventional/shallow classifier is utilized, and this classifier is named Fine kNN. The hyperparameters of the classifier are tabulated in Table 4.

Step 8: Calculate the results of the selected most appropriate feature vector deploying two validations. The validations used were 10-fold cross-validation and LOSO cross-validation.

#### 2.2.4. Iterative Hard Majority Voting

Iterative hard majority voting [40] is the last phase of the presented CGP17Pat-based schizophrenia detection model. The used data set had 19 channels. The calculated prediction labels from each channel were used to calculate general results. They were qualified according to their classification accuracy. The qualified predicted labels were voted using the mode function. In this work, iteration was initialized from 3 (three channels) to 19. Finally, the best classification accuracy was chosen using a greedy search.

Step 9: Apply iterative hard majority voting to the generated 19 predicted labels from 19 channels using kNN.

## 3. Results

The performance of the presented CGP17Pat-based schizophrenia detection model is evaluated in this section.

### 3.1. Experimental Setup

The proposed parametric CGP17Pat-based EEG classification model used in this study contains four phases. To implement the CGP17Pat-based EEG classification model, a MATLAB (2021b) environment was used. The parameters used in this EEG classification model are listed in Table 5.

### 3.2. Performance Metrics

By deploying the parameters above (Table 5), the proposed CGP17Pat-based schizophrenia detection model was implemented using a MATLAB (2021b) environment. The model’s accuracy (acc), sensitivity (sen), specificity (spe), and geometric mean (gm) were calculated. Mathematical notations of the performance metrics are given in Equations (11)–(14) [41,42]:

(11) $$acc=\frac{tp+tn}{tp+fn+tn+fp}$$

(12) $$sen=\frac{tp}{tp+fn}$$

(13) $$spe=\frac{tn}{tn+fp}$$

(14) $$geo=\sqrt{spe\times sen}$$

Herein, tp, fn, tn, and fp are the number of true positives, false negatives, true negatives, and false positives.

### 3.3. Performance Evaluation

The results were calculated using 10-fold cross-validation and LOSO. Moreover, this data set contained 19 channels, and the channel-wise (channel by channel) results are listed in Table 6.

The best results are noted in bold font type. According to the results in Table 6, the best accurate channel was Pz based on the 10-fold cross-validation, and our proposed model reached a 99.82% classification accuracy and geometric mean. Furthermore, our model yielded an 82.40% classification accuracy using LOSO validation on the F7 channel.

The EEG data set had 19 channels, and the channel-wise results were also calculated using the presented CGP17Pat-based EEG signal classification model. To calculate the general (channel-wise) results, iterative hard majority voting was applied to the prediction vectors. The calculated voted results are tabulated in Table 7.

Table 7 shows that iterative hard majority voting algorithm increased classification accuracy from 99.82% to 99.91% for the 10-fold cross-validation and from 82.40% to 84.33% for the LOSO validation.

**Figure 4:**
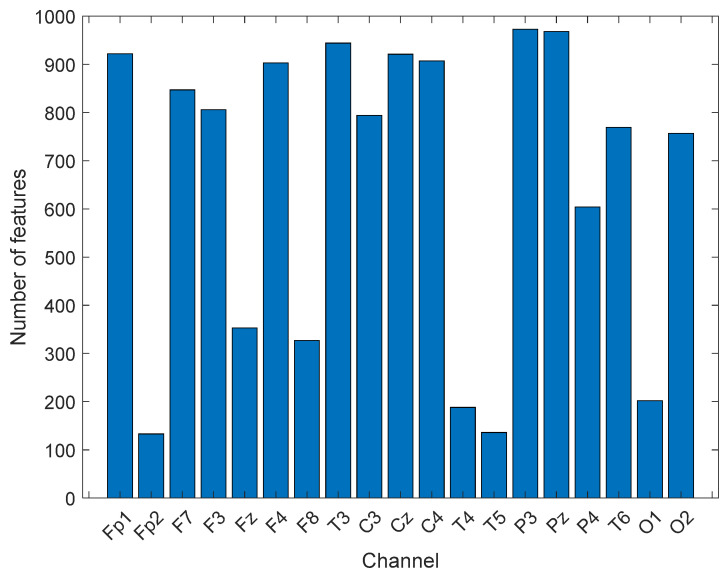
The lengths of the optimal feature vectors chosen by INCA.

CGP is the most widely used mathematical model to create cyclic groups for cryptographic applications. Here, CGP was utilized to propose a new generation nonlinear pattern. By using 17 as modulo, eight cyclic groups were created. Each group’s creation was considered to create a center symmetric local feature extractor. The presented CGP17Pat created 2048 features. The most valuable feature vector was chosen using the INCA selector. The range of the length of the selected optimal feature vectors was [133–973]. INCA chose 133 features for the Fp2 channel, and 973 features for the P3 channel. The number of the selected features from each channel chosen by INCA are depicted in Figure 4.

These feature vectors were then classified using kNN. By using the kNN classifier, the results of all channels were calculated. Iterative hard majority voting was used to calculate the general classification results, and 99.91% and 84.33% classification accuracies were obtained using 10-fold cross-validation and LOSO cross-validation, respectively.

The second evaluation parameter was time/computational complexity. Big O notation was used to calculate the time complexity of our proposed CGP17Pat-based model, and the phase-by-phase results are given below.

*Feature extraction:* In this phase, a decomposition model and CGP17Pat feature generation function were used. The time burden of the CGP17Pat was equal to O(8n)≅O(n). Furthermore, this feature extractor (CGP17Pat) generates features from decomposed signals. Therefore, O(ndlognd) is calculated as the time complexity of the CGP17Pat-based multilevel feature extraction method. Here, n represents the length of the signal, and d defines the number of instances.

*Feature selection:* In the feature chosen phase, the INCA function was used, and it uses two parameters: loop range and loss function. Moreover, NCA was applied to calculate the indexes qualified of the features. Considering these parameters, the complexity of the INCA was calculated as O(td+lnd). Herein, t is the complexity coefficient of the NCA, and l defines the number of feature vectors.

*Classification:* kNN was employed to obtain the classification results. The time complexity of the kNN is O(nd).

The computational/time complexity of this model is equal to O(ndlognd+td+lnd+nd)≅O(ndlognd+td+lnd). This result demonstrates that our proposed CGP17Pat-based schizophrenia classification model has linear complexity. Therefore, this model is a lightweight classification model.

## 4. Discussion

**Figure 5:**
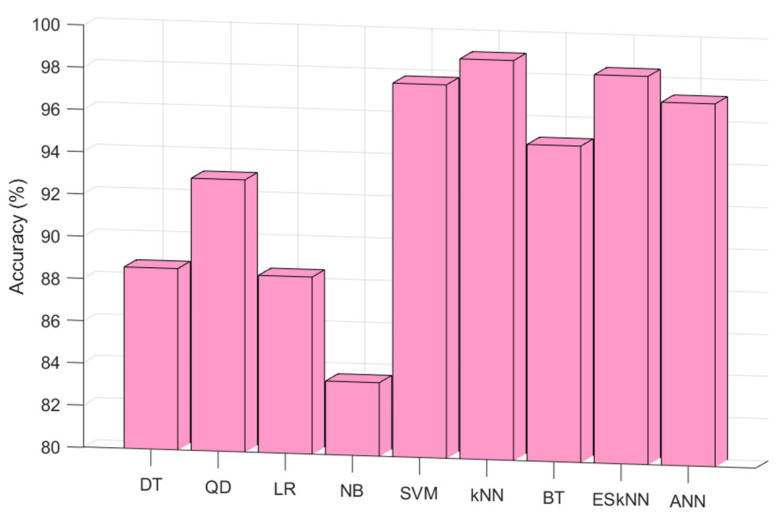
Classification accuracies of the decision tree (DT), quadratic discriminant (QD), logistic regression (LR), naive Bayes (NB), support vector machine (SVM), Fine kNN (kNN), bagged tree (BT), ensemble subspace kNN (ESkNN), and artificial neural network (ANN) for the Fp2 channel with 10-fold cross-validation.

In this work, a hand-crafted feature extraction function (CGP17Pat)-based EEG signal classification model was presented to detect schizophrenia automatically. The proposed hand-modeled learning method uses kNN as both the INCA classifier and loss value generator. This classifier was selected according to the experiments. According to the results of the shallow classifiers (testing results), the best classifier was Fine kNN. The test results of the tested classifier on the Fp2 by employing a 10-fold cross-validation are depicted in Figure 5.

**Figure 6:**
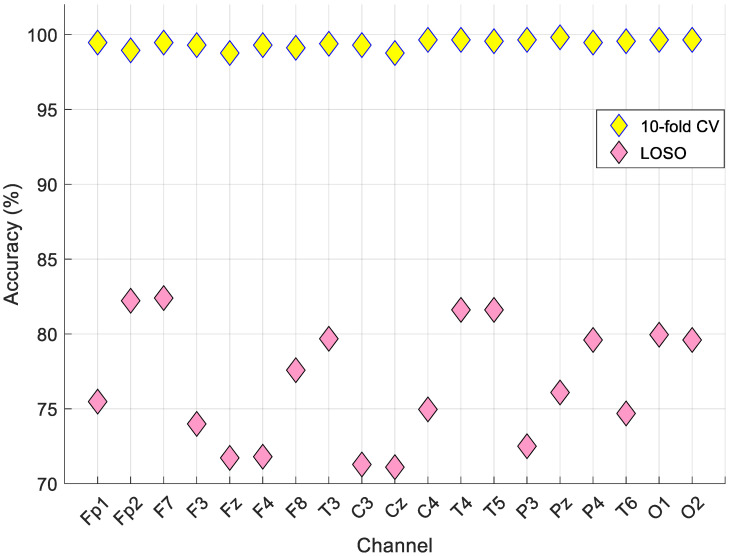
The obtained comparative results according to the validation technique.

From Figure 4, the best classifier was weighted kNN for this data set. Therefore, kNN was used as the classifier in this research. Moreover, two validation techniques were used, and they were 10-fold cross-validation and LOSO cross-validation. The classification results according to the validation techniques are shown in Figure 6.

**Figure 7:**
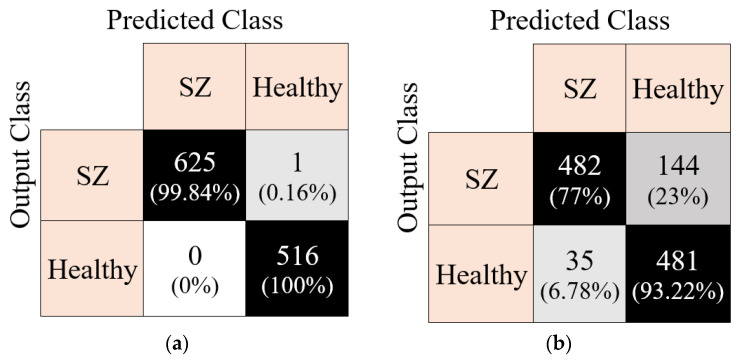
Voted results: confusion matrices of the presented CGP17Pat-based EEG classification model using (**a**) 10-fold cross-validation and (**b**) LOSO cross-validation.

From Figure 6, the best accurate validation technique was the 10-fold cross-validation. This model attained the best results on the Pz channel for 10-fold cross-validation and the F7 channel for LOSO validation. Iterative hard majority voting was applied to these results, and the general results calculated are denoted in Figure 7 using confusion matrices.

To denote the success of the presented model, we compared our results with other machine learning models for the automatic detection of schizophrenia reported from 2019 to 2021 as tabulated in Table 8.

As observed from Table 8, Shoeibi et al. [49] attained 99.25% accuracy using five-fold cross-validation when CNN and LSTM were used. Oh et al. [21] presented a CNN-based model, where they calculated both subject-wise and non-subject-wise results. An accuracy of 98.07% was obtained using 10-fold cross-validation and 81.26% with LOSO cross-validation. Shoeibi et al. [49] and Oh et al. [21] used deep learning to attain high classification accuracies. Our CGP17Pat-based model, on the other hand, achieved the highest classification accuracy of 99.91% for 10-fold cross-validation and 84.33% with LOSO validation. Furthermore, our presented model is a lightweight EEG classification method, where the time complexity of the CGP17Pat is O(n) and the time burden of the presented multilevel CGP17Pat-based feature extraction method is O(nlong). Since a hand-modeled classification method was used here, parameter tuning was not required. Baygin [46] presented a statistical model to automatically detect SZ and attained over 99% classification accuracy deploying 10-fold cross-validation. However, there were no results on LOSO cross-validation in the paper. In summary, the presented CGP17Pat-based EEG signal classification model attained the best classification accuracy among the available machine learning methods with 10-fold cross-validation.

The benefits and limitations of the work are discussed below.

Benefits:

- A cryptographic model (CGP) was used, where CGP was used for its feature extraction ability;
- A simple machine learning model was presented using the presented CGP17Pat;
- A hand-modeled EEG signal classification model was proposed with low time complexity. Only CGP17Pat was used to extract the salient features, and the time complexity of this function is *O(nlong)* according to Big O notation;
- LOSO and 10-fold cross-validations were used to depict the robustness of this model;
- To denote the feature generation capability of the CGP17Pat, a shallow classifier was used, and high accuracy values were obtained.

Limitations:

- Using LOSO validation, the presented model (i.e., CGP17Pat-based classification model) attained unsatisfactory results for several channels (especially Fz, F4, C3, and Cz);
- We used a hand-modeled learning technique, but INCA had high time complexity;
- The hyperparameters of the kNN can be optimized.

The proposed model can be used in psychiatry clinics to detect schizophrenia using EEG signals, and we also intend to use this model to detect different types of schizophrenia to help clinicians in their treatment.

## 5. Conclusions

We presented a new feature extraction function using a cryptologic method, which we named CGP17Pat. The CGP17Pat function was used to classify EEG signals for automatic detection of SV. The model attained high accuracies of 99.91% and 84.33% with 10-fold cross-validation and LOSO cross-validation, respectively. The results demonstrated the excellent feature classification ability using CGP17Pat. By deploying LOSO cross-validation, the real-world performance of the proposed CGP17Pat-based model was simulated, and comparable results were obtained using 10-fold cross-validation. This model can assist psychologists/psychiatrists in their diagnosis of schizophrenia so that early treatment can be provided to affected patients.

Our future plan is to develop an automated detection application for various mental disorders using EEG signals. These signals collected from worldwide medical centers will be fed into our smart system. As a result, the computerized system will diagnose the different mental disorders quickly and accurately so that the medical professionals can provide the necessary treatment and intervention to help their patients. Moreover, many feature extraction functions can be presented using CGP with other modulo values and another new CGP-based deep learning model can be developed in the near future.

## Figures and Tables

**Table 1:** Automatic SZ classification using ML techniques.

Author(s)	Year	Data Set Feature	Method
R. Buettner et al. [17]	2020	EEG [18,19];	Spectral analysis-based feature extraction and classification using random forest.
		14 schizophrenias, 14 HCs;	
		1 min segmentation.	
V. Jahmunah et al. [20]	2019	EEG [18,19];	Nonlinear statistical moment-based feature extraction, feature selection with *t*-tests, and classification using SVM.
		14 schizophrenias, 14 HCs;	
		25 s segmentation.	
S.L. Oh et al. [21]	2019	EEG [18,19];	Convolutional neural network
		14 schizophrenias, 14 HCs;	
		25 s segmentation.	
L.S. Mayo et al. [22]	2017	EEG;	Feature extraction at time and frequency domains, J5 feature selection, and classification with multilayer perceptron.
		16 schizophrenias, 31 HCs;	
		0.8 s segmentation.	
L. Zhang [23]	2019	EEG [24];	Event-related potential feature extraction and classification with random forest.
		49 schizophrenias, 32 HCs;	
		3 s segmentation.	
Z. Chen et al. [25]	2020	Magnetic resonance images (MRIs) [26];	Image segmentation for detecting gray matter, white matter, and cerebrospinal fluid; two-sample *t*-test-based feature selection; classification with SVM.
		34 schizophrenias, 34 HCs.	
C.W. Espinola et al. [27]	2021	Voice;	Acoustic feature extraction, particle swarm optimization (PSO)-based feature selection, and classification using SVM.
		20 schizophrenias, 11 HCs;	
		96.9 min HC, 125.7 min schizophrenia	
		10 s segmentation.	
A.N. Chandran et al. [28]	2021	EEG [18,19];	Time-domain-based feature extraction and classification deploying long short-term memory (LSTM).
		14 schizophrenias, 14 HCs;	
		4 s segmentation	
D. Lei et al. [29]	2019	MRIs	Gray matter, white matter, low-frequency fluctuation, regional homogeneity, structural covariance matrices, and functional connectivity matrices and SVM classifier.
		Combination of five data sets;	
		295 schizophrenias, 452 HCs;	
H. Akbari et al. [30]	2021	EEG [18,19];	Graphical feature extraction, forward feature selection algorithm, and classification with kNN.
		14 schizophrenias, 14 HCs;	
Z. Aslan and M. Akin [31]	2020	Two EEG data sets:	Spectrogram images from EEG signals and classification using VGG16 deep network.
		Data set 1: 45 schizophrenias, 39 HCs [32],	
		Data set 2: 14 schizophrenias, 14 HCs [18,19];	
		5 s segmentation.	

**Table 2:** Attributes of the data set used in this work.

Feature	Value
Groups	14 Schizophrenic groups, 14 control groups
Gender	28 Patients (14 males, 14 females)
Average Age	27.9 ± 3.3 (7 schizophrenic males)
	28.3 ± 4.1 (7 schizophrenic females)
	26.8 ± 2.9 (7 healthy males)
	28.7 ± 3.4 (7 healthy females)
Length of Each EEG Segment	25 s (250 × 25 = 6250)

**Table 3:** Generated cyclic group of 17 orders.

**G1**	3	9	10	13	5	15	11	16	14	8	7	4	12	2	6	1
**G2**	5	8	6	13	14	2	10	16	12	9	11	4	3	15	7	1
**G3**	6	2	12	4	7	8	14	16	11	15	5	13	10	9	3	1
**G4**	7	15	3	4	11	9	12	16	10	2	14	13	6	8	5	1
**G5**	10	15	14	4	6	9	5	16	7	2	3	13	11	8	12	1
**G6**	11	2	5	4	10	8	3	16	6	15	12	13	7	9	14	1
**G7**	12	8	11	13	3	2	7	16	5	9	6	4	14	15	10	1
**G8**	14	9	7	13	12	15	6	16	3	8	10	4	5	2	11	1

**Table 4:** Fine-tuned hyperparameters of the kNN classifier.

Hyperparameter	Value
*k*	1
Distance	Euclidean
Weight	None
Standardize Data	True

**Table 5:** Parameters of our CGP17Pat-based EEG classification model.

Phase	Parameters
Feature Extraction	MAP: 2-, 4-, and 8-sized overlapping blocks were used
	CGP17Pat: 16-sized overlapping blocks were used, and eight patterns were deployed
INCA	Range: [100–1000] Error function: kNN
Classification	Fine kNN with 10-fold cross-validation and LOSO
Iterative Hard Majority Voting	The iteration range selected was [3,4,5,6,7,8,9,10,11,12,13,14,15,16,17,18,19]

**Table 6:** The 10-fold cross-validation and LOSO cross-validation results (%) of the CGP17Pat-based model.

Channel	10-Fold Cross-Validation				LOSO Cross-Validation			
	Accuracy	Sensitivity	Specificity	Geometric Mean	Accuracy	Sensitivity	Specificity	Geometric Mean
Fp1	99.47	99.36	99.61	99.49	75.48	69.65	82.56	75.83
Fp2	98.95	99.04	98.84	98.94	82.22	80.03	**84.88**	82.42
F7	99.47	99.20	99.81	99.50	**82.40**	**80.83**	84.30	**82.55**
F3	99.30	99.20	99.42	99.31	73.99	69.49	79.46	74.31
Fz	98.77	98.56	99.03	98.80	71.72	70.13	73.64	71.86
F4	99.30	99.52	99.03	99.28	71.80	66.77	77.91	72.13
F8	99.12	99.36	98.84	99.10	77.58	77.96	77.13	77.54
T3	99.39	99.20	99.61	99.41	79.68	80.51	78.68	79.59
C3	99.30	99.04	99.61	99.33	71.28	77.48	63.76	70.28
Cz	98.77	98.72	98.84	98.78	71.10	68.05	74.81	71.35
C4	99.65	99.52	99.81	99.66	74.96	69.17	81.98	75.30
T4	99.65	99.52	99.81	99.66	81.61	82.27	80.81	81.54
T5	99.56	99.36	99.81	99.58	81.61	80.67	82.75	81.70
P3	99.65	99.52	99.81	99.66	72.50	73.80	70.93	72.35
Pz	**99.82**	**99.84**	99.81	**99.82**	76.09	80.35	70.93	75.49
P6	99.47	99.52	99.42	99.47	79.60	77.80	81.78	79.76
T6	99.56	99.68	99.42	99.55	74.69	71.73	78.29	74.94
O1	99.65	99.36	**100**	99.68	79.95	77.48	82.95	80.16
O2	99.65	99.52	99.81	99.66	79.60	78.12	81.40	79.74

**Table 7:** The calculated voted results (%) according to the 10-fold cross-validation and LOSO cross-validation.

Validation	Number of Channels	Accuracy	Sensitivity	Specificity	Geometric Mean
10-fold	3	99.91	99.84	100	99.92
LOSO	17	84.33	77	93.22	84.72

**Table 8:** Automatic schizophrenia classification based on EEG signals (2019–2021).

Author(s)	Year	Method	Segmentation	Validation	Result(s)
R. Buettner et al. [17]	2020	Spectral analysis, random forest	1 min	10-Fold cross-validation	Acc. = 96.77
					Bac. = 96.77
					Kap. = 93.55
R. Buettner et al. [43]	2019	Independent component analysis, random forest	-	10-Fold cross-validation	Acc. = 71.43
					Bac. = 80.0
P.T. Krishnan et al. [44]	2020	Multivariate empirical model decomposition, entropy computation, recursive feature elimination, and SVM	2 s	10-Fold cross-validation	Acc. = 93.0
					Auc. = 98.31
					Sen. = 94.0
					Spe. = 92.0
					Pre. = 92.71
					FScr. = 93.04
A.N. Chandran et al. [28]	2021	Time-domain feature extraction, long short-term memory (LSTM)	4 s	Holdout	Acc. = 99.0
					Pre. = 99.2
				88:12	Rec. = 98.9
					FScr. = 99.0
K. Singh et al. [45]	2021	Fast Fourier transform, spectral feature extraction, CNN, and LSTM	5 s	Holdout	Acc. = 98.96
					Sen. = 99.05
				90:10	Spe. = 98.88
					FScr. = 98.87
S.L. Oh et al. [21]	2019	Custom convolutional neural network (CNN) design, subject and non-subject based testing	25 s	10-Fold cross-validation	Non-Sub.
					Acc. = 98.07
					Sen. = 97.32
					Spe. = 98.17
					Subject
					Acc. = 81.26
					Sen. = 75.42
					Spe. = 87.59
M. Baygin [46]	2021	Tunable Q-factor wavelet transform (TQWT), statistical moment, ReliefF, and kNN	25 s	10-Fold cross-validation	Acc. = 99.12
					Pre. = 99.04
					Rec. = 99.36
					Geo. = 99.10
					FScr. = 99.20
K. Kim et al. [47]	2021	Microstate features; statistical, frequency, and time domain features; *t*-test; recursive feature elimination; SVM	5 s	10-Fold cross-validation	Acc. = 75.64
					Auc. = 80.19
					Sen. = 71.93
					Spe. = 75.50
M. Krishnaveni et al. [48]	2019	Non-local mean algorithm, empirical mode decomposition, discrete Fourier transform, mel-warp triangular filter, and optimized backpropagation neural network	-	10-Fold cross-validation	Acc. = 90.26
					Sen. = 88.64
					Spe. = 89.17
V. Jahmunah et al. [20]	2019	Nonlinear feature extraction, *t*-test, and SVM	25 s	10-Fold cross-validation	Acc. = 92.91
					Sen. = 93.45
					Spe. = 92.24
H. Akbari et al. [30]	2021	Graphical feature extraction, forward selection algorithm, and kNN	-	10-Fold cross-validation	Acc. = 94.80
					Sen. = 94.30
					Spe. = 95.20
Z. Aslan and M. Akin [31]	2020	Spectrogram images from EEG signals, VGG16-based CNN	5 s	-	Acc. = 97.0
					Rec. = 97.0
					FScr. = 97.0
A. Shoeibi et al. [49]	2021	CNN and LSTM	25 s	5-Fold cross-validation	Acc. = 99.25
					Pre. = 98.33
					Rec. = 98.86
					Auc. = 99.73
M. Sharma and U.R. Acharya [50]	2021	L1 Norm, ES-KNN	25 s	10-Fold cross-validation and LOSO	10-fold CV
					Acc. = 99.21
					LOSO CV
					Acc. = 97.2
Our Method		CGP17Pat, MAP, INCA, kNN, and iterative hard majority voting	25 s	10-Fold cross-validation and LOSO	10-fold CV
					Acc. = 99.91
					LOSO CV
					Acc. = 84.33

Acc. = accuracy; Sen. = sensitivity; Spe. = specificity; Bac. = balanced accuracy; Kap. = kappa; FScr. = F-score; Pre. = precision; Rec. = recall; Geo. = geometric mean; SVM = support vector machine; kNN = k-nearest neighbor; CV = cross-validation.

## Data Availability

Not applicable.
